# Design of 3D Additive Manufactured Hybrid Scaffolds for Periodontal Repair Strategies

**DOI:** 10.1021/acsabm.5c00561

**Published:** 2025-07-31

**Authors:** Valentina Peluso, Roberto De Santis, Antonio Gloria, Giusy Castagliuolo, Anna Zanfardino, Mario Varcamonti, Teresa Russo

**Affiliations:** † Institute of Polymers, Composites and Biomaterials, 9327 National Research Council of Italy, 80125 Naples, Italy; ‡ Department of Biology, 9307 University of Naples Federico II, 80126 Naples, Italy; § Department of Industrial Engineering, 9307 University of Naples Federico II, 80125 Naples, Italy

**Keywords:** design for additive manufacturing, hybrid structure, morphological analyses, mechanical measurements, antimicrobial activity, dental, oral, and craniofacial tissue repair

## Abstract

*Background*: Nonconventional fabrication technologies (*e.g.*, additive manufacturing and 3D bioprinting) represent a challenging approach to the design of 3D scaffolds as extracellular matrix analogues with appropriate properties for supporting cell behavior over time. *Methods*: A strategy to develop 3D additive manufactured hybrid scaffolds with dual porosity and tailored morphological and mechanical/functional features was proposed *via* the combination of synthetic (poly-ε-caprolactone) and natural (chitosan) polymers. Design of 3D additive manufactured hybrid scaffolds, morphological analysis, *in vitro* swelling and degradation measurements, mechanical measurements, antimicrobial assays against both oral cavity-specific and nonoral bacteria, and biological assays using periodontal ligament stem cells (PDLSCs) or human osteosarcoma cells (MG63) were carried out. *Results*: The inclusion of the chitosan network improves the dimensional stability of the structure as well as the cell retention effect, ensuring antimicrobial activity. *Conclusion*: The current study represents a first step for future complex works with the aim of studying the effect of the inclusion of chitosan network in a 3D porous multifunctional structure obtained *via* additive manufacturing technologies, also taking into account the possibility of modulating the mechanical behavior, adopting two different swelling and degradation rates in order to tune drug/protein/gene delivery over time, and thus tailoring the tissue regeneration process and the health of the oral microbiota.

## Graphical abstract

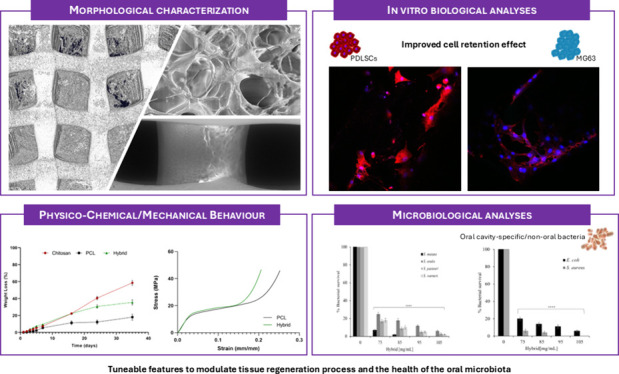

## 1 Introduction

Biomimetic devices designed to repair or replace damaged tissues and organs—while integrating a multidisciplinary perspective spanning from medicine, biology, chemistry, engineering, and life sciences—still represent a significant challenge for modern science.^1–4^ The ability to design and fabricate 3D scaffolds as analogues of the extracellular matrix opens new scenarios in scaffold development and optimization strategies, aiming to provide structural support for cell adhesion and tissue regeneration in a tissue-specific manner.^5^

Despite notable progress in the repair of simple tissue defects, the regeneration of complex tissues continues to demand significant effort from the scientific community.^6^

Scaffolds for tissue-engineering applications are generally expected to meet several key criteria: (i) biocompatibility; (ii) the ability to remodel in accordance with the rate of tissue regeneration or repair; (iii) a partially or fully interconnected porous structure to support cell infiltration and ensure the transport of nutrients and metabolic waste; (iv) surface features conducive to cellular functions. Additionally, (v) mechanical stability and ease of sterilization are essential for surgical handling. Furthermore, the incorporation of functional chemical cues represents a promising strategy for the controlled release of bioactive molecules—such as growth factors, cytokines, drugs, or genes—transforming the scaffold into a bioactive reservoir.

In recent years, various strategies for periodontium regeneration have been proposed, particularly combining 3D scaffolds with cell-based approaches employing periodontal ligament stem cells (PDLSCs).^7^ Multilayered or multicomponent scaffolds capable of providing the physiological and mechanical support required for the regeneration of this complex tissue continue to represent a significant challenge.^7^ Currently, the ultimate goal of periodontal therapy is to restore an intact and functional periodontium, similar to its healthy state, following the elimination of the bacterial cause of the disease.^8^ If left untreated, periodontal tissues may deteriorate, leading to tooth loss. Moreover, the inflammatory processes associated with periodontitis may trigger microbial dysbiosis and contribute to a destructive inflammatory state.^9^ Recent studies suggest a potential link between oral and gut microbiota dysregulation and Alzheimer’s disease (AD). Alterations in the microbial composition have been observed in patients with subjective or mild cognitive impairment, as well as in those with AD dementia.^10^ There is growing evidence that pathogenic bacteria from dental plaque may enter the bloodstream, traverse the blood–brain barrier—especially when its integrity is compromised—and reach the brain, where they promote the expression of inflammatory cytokines and vascular adhesion molecules.^11^ The first evidence of periodontal bacterial infection leading to hippocampal damage has been recently reported.^12^ Additionally, several oral bacterial strains (*e.g.*, ,, , , , and ) have been demonstrated to be involved in inflammatory diseases at remote organ sites like AD,^13^ considering that periodontitis-related bacterial strain and their related gingipains have been frequently detected in autopsy brain tissues of AD patients, while the same scenario has not been found in health human brain.^14^

Anyway, the pathophysiological mechanisms that link AD onset and periodontitis remain poorly investigated, although it is frequently reported that the treatment of inflammatory states related to periodontitis represents a crucial aspect for both oral and general health.

Over the past two decades, a wide range of regenerative periodontal therapies have been explored, taking into account the complexity of the periodontium as the multicomponent tooth-supporting tissue composed of gingiva, cementum, periodontal ligament (PDL), and alveolar bone.

To date, the possibility to regenerate these complex damaged tissues still represents an unmet objective.^8^

Multimaterial and multilayered scaffolds with tailored features in each layer are required for bioinspired design strategies focused on periodontal structures, thus triggering materials and functional features of each compartment in terms of morphological and chemical cues, as well as cellular/biochemical composition toward periodontal complex regeneration.^15^

Advanced fabrication techniques, such as “additive manufacturing” (AM) or “3D printing” enable the production of structures in a layer-by-layer fashion, producing customized scaffolds with reproducible inner morphology and complex shape, starting from a 3D model or a CAD (Computer-Aided Design) model, which can be obtained either by specific modeling software or derived from medical imaging data, such as computed tomography or magnetic resonance imaging.^16^

3D printing has been proposed for microgrooved and microchannelled structured scaffolds to guide fiber orientation,^17^ benefiting from the possibility to customize the scaffold according to the patient needs.^18^

The introduction of microfabrication techniques has also allowed to tailor the micropattern shape and size for aligned tissue formation,^19^ thus influencing cell orientation according to the adopted lay-down pattern for scaffold fabrication.^16^

Synthetic polymers [*e.g.*, poly­(caprolactone) (PCL), poly­(glycolic acid) (PGA), poly­(L-lactic acid) (PLLA), or poly­(lactic-*co*-glycolic) acid (PLGA)] still remain among the widely adopted for 3D periodontal scaffold fabrication and have been extensively investigated over the past decade.^20,21^ Natural polymers such as collagen, gelatin, and chitosan (CS) have been often employed as injectable material for topical administration of drugs or growth factors and combined with synthetic polymers, thus improving the hydrophilicity and tailoring degradation rate and drug/protein/gene delivery.

Among others, CS represents a promising material for periodontal therapies due to its ability to stimulate cell proliferation and osteogenesis, as well as for its antioxidant, antimicrobial, and hemostatic activities.^22^

Multimaterial or hybrid scaffolds^23,24^ that are able to integrate the best properties of both synthetic and natural polymers and characterized by a tailored structure porosity may play a pivotal role in the development of the next generation of 3D scaffolds for tissue engineering. Surface topography, pore size and geometry, porous networks, and macroscopic pore arrangement represent four different levels of features for tissue-engineering scaffolds, introduced by Gariboldi and Best in 2015.^25^

Anyway, over the past years, great efforts have been devoted to the development of devices with optimized features for different kinds of applications, benefiting from the advances in methodologies and design strategies.^21^ Furthermore, biomimetic high-strength biomechanical implants with optimized geometry and mass-transport properties have been widely investigated in recent literature^26^ also introducing an intriguing strategy toward the development of multifunctional 3D hybrid structures and their eventual combination with active antibacterial systems and magnetic stimulation for tissue prevention, treatment, or repair.^27^

Tissue-engineered constructs hold promise for regenerating the bone-ligament complex in cases of periodontal damage caused by disease or trauma.^28^ Acute periodontal bone defect using a patient-specific, bioresorbable, 3D-printed polymer scaffold combined with a signaling growth factor has been recently documented with a 12-month follow-up period.^29^ The reported findings highlight the potential of image-guided 3D-printed scaffolds for periodontal regeneration, combined with systemic antibiotics administration. Despite the widespread use of PCL in bone tissue engineering, its inherent hydrophobicity and lack of specific cell recognition sites limit its biological performance. To overcome these drawbacks, Dong et al.^23^ developed a hybrid scaffold by incorporating an injectable, thermosensitive CS hydrogel into a 3D-printed PCL scaffold. This study investigates the potential of the CS/PCL hybrid system to enhance the cell seeding efficiency, osteoinductivity, and mechanical properties. Rabbit bone marrow mesenchymal stem cells and bone morphogenetic protein-2 were encapsulated within the hydrogel to evaluate the cellular responses and osteogenic potential. Compared with pure PCL scaffolds, the hybrid system demonstrated improved cell proliferation, viability, and osteogenic differentiation, suggesting the use of the proposed scaffolds for bone and subchondral defect repair.

The study herein proposed pioneers the design and fabrication of innovative 3D hybrid scaffolds through AM techniques, aiming to combine high mechanical performance with excellent cell compatibility. By strategically integrating traditional fabrication techniques with AM technologies and by merging distinct polymer sources (CS and PCL), we developed structurally robust scaffolds with significant potential for tissue-engineering applications. A comprehensive characterization was carried out, including morphological analyses, *in vitro* swelling and degradation assessments, mechanical measurements, and antimicrobial evaluations against both oral cavity-specific and non-oral pathogens. Furthermore, biological performance was assessed by adopting two relevant cell models: human PDLSCs and the osteosarcoma-derived MG63 cell line, both seeded onto the engineered scaffolds. PDLSCs, recently recognized as a promising tool in regenerative dentistry and tissue engineering, were chosen due to their accessibility, multilineage differentiation capabilities (osteogenic, chondrogenic, and adipogenic), and immunomodulatory properties, comparable to bone marrow and dental pulp mesenchymal stem cells.^30–38^ In parallel, MG63 cells, widely regarded as reliable *in vitro* “bone mimics,”^39^ offered a standard model to evaluate osteogenic compatibility.

The combination of advanced materials, hybrid fabrication strategies, and comprehensive biological validation demonstrates the strong potential of these scaffolds for applications in periodontal regeneration and bone tissue reconstruction. In particular, antimicrobial and biological assays revealed encouraging results, further supporting their applicability in clinical contexts requiring both structural support and regenerative functionality.

This study would also lay the groundwork for application in the treatment of periodontal diseases potentially linked to the onset of AD, due to the antimicrobial features of the provided structures against oral cavity strains and periodontal pathogen, with the possibility to locally target the bacteria and lower the release of their relative virulence factors, including gingipains, which are implicated in AD.^9^

## 2 Materials and Methods

### 2.1 3D Polysaccharide-Based Scaffolds

CS scaffolds were obtained according to a previously reported procedure. Briefly, a viscous CS solution was obtained by dissolving CS [CAS 9012-76-4, molecular weight of ∼300 kDa, deacetylation degree 85%, Heppe Medical Chitosan GmbH, Halle, Germany] in 1% (v/v) acetic acid (Sigma, Milan, Italy) at room temperature and stirring overnight (4000 rpm). An adequate volume (10–30 μL) of the dibasic sodium phosphate (Na_2_HPO_4_, Sigma, Milan, Italy) solution at 100 mg/mL concentration was added dropwise into the CS solution under magnetic stirring until the mixture reached a pH of 7.0–7.2. Finally, the autogelling solutions were transferred into a 48-well plate and incubated at 37 °C overnight. The obtained CS hydrogel was stored in a refrigerator at −80 °C for 24 h and lyophilized in a freeze-dryer for 36 h at *T* = −52 °C and *P* = 1 Pa.

### 2.2 Design of 3D Additive Manufactured Scaffolds

Neat PCL scaffolds (10 mm in length, 10 mm in width, and 3 mm in height) with controlled and interconnected porosity were fabricated by processing PCL (*M*_n_ = 45,000, Sigma-Aldrich) pellets through the 3D fiber deposition technique, adopting an Allevi 3 3D Bioprinter (Allevi Inc., Philadelphia, PA, USA). The printing parameters are reported in Table 1.

**Table 1:** Printing Parameters^a^

LH [μm]	PS [mm/s]	ID [mm]	PT [°C]	*P* [PSI]
300	3	1.6	110	20

a Layer height (LH), infill distance (ID), printing speed (PS), printing temperature (PT), and operative pressure (*P*).

A nozzle with an inner diameter of 400 μm was used, obtaining square pores of 300 μm compatible with scaffold porosity for hard tissue regeneration, as frequently reported in the literature.^40^

### 2.3 Design of 3D Additive Manufactured Hybrid Scaffolds

Hybrid PCL/CS scaffolds were obtained by infiltrating the 3D additive manufactured PCL structures with the CS solution, promoting the gelation according to the procedure described in Section 2.1. After infiltration with autogelling CS solution, samples were incubated at 37 °C overnight for gelling the CS inside the pores of the scaffolds; samples were subsequently stored in a refrigerator at −80 °C for 24 h and lyophilized in a freeze-dryer for 36 h at *T* = −52 °C and *P* = 1 Pa, thus obtaining structures characterized by a -pore network in which the smaller pore structure emulates a vascular network, and it should be useful in enhancing initial cell adhesion and mass-transport properties.^40^

### 2.4 Morphological Analyses

Scanning electron microscopy (SEM, FEI inspect SEM) was used to investigate the morphology of the neat PCL scaffolds, CS porous structures and PCL/CS hybrid scaffolds, focusing on the smaller pores and the correct CS infiltration into the 3D additive manufactured PCL scaffolds.

The 3D structure and pore architecture were qualitatively and quantitatively investigated via X-ray computed tomography (XCT) analyses performed using the Nano3DX tomograph (Rigaku Corp., Japan) equipped with a Cr anode operating at 35 kV, 25 mA. All samples were analyzed by adopting different resolution lenses and parameters as reported in Table 2. The volume reconstruction was carried out with the proprietary software Nano3DX reconstruction as well as adopting ImageJ for image processing of the cross sections obtained via XCT measurements, according to previous works.^41–43^ In particular, the manipulation of the grayscale thresholds enabled us to finely remark the boundaries of the 3D porous scaffold, as the negative space representative of the pore volume fraction, thus evaluating the characteristic parameters such as the structural porosity, pore shape, and size.

**Table 2:** Summary of Operative Parameters Used for XCT Analysis

field of view (FOV, mm^2^)	acquisition resolution (μm/pixel)	X-ray detector position (mm)	exposure time (s)	range of sample rotation angle (deg)	number of projections	scan time (min)
2.662 × 2.662	1.3	2	5	180	600	70
10.65 × 10.65	5.2	2	5	180	600	70

### 2.5 Mechanical Measurements

The mechanical performances of the developed structures were evaluated by compression tests. An INSTRON 5566 testing machine was used at a crosshead speed of 1 mm/min and up to a maximum stress of 50 MPa. The engineering stress (σ) and strain (ε) were evaluated through the following expressions (eqs 1 and 2):^44^

$$\sigma =\frac{F}{L\cdot W}$$ 1

$$\varepsilon =\frac{\Delta H}{H_{0}}$$ 2

where *F* is the force, as measured by the load cell, and Δ*H* represents the variation of the scaffold height.

The compressive modulus of the samples was determined by considering the slope of the initial and linear portion of the stress–strain curve. For statistical purposes, the experiments were conducted in triplicate.

### 2.6 *In Vitro* Degradation

Water uptake and weight loss measurements were carried out on dried CS cylindrical scaffolds (13 mm in diameter, 13 mm in height) and hybrid scaffolds (10 mm × 10 mm × 3 mm) immersed in 4 mL of PBS at 37 °C. At defined time points, the swollen structures were weighed after removal of excess PBS via filter paper. Meanwhile for weight loss measurements, scaffolds were frozen (−86 °C) and lyophilized to evaluate the specific weight after a defined incubation time.

Water uptake (WU) and weight loss (WL) have been evaluated according to eqs 3 and 4, respectively:^23^

$$\mathrm{WU}=\frac{W_{t}-W_{0}}{W_{0}}\%$$ 3

$$\mathrm{WL}=\frac{W_{\mathrm{eq}}-W_{t}}{W_{\mathrm{eq}}}\%$$ 4

The equilibrium weight was reported as *W*_eq_; *W_t_* is the sample weight at each time point, while *W*_0_ represents the dry sample weight (*t* = 0). Data have been reported as the mean value ± standard deviation. Measurements were performed at least in triplicate.

### 2.7 Microbiological Analyses

#### 2.7.1 Bacterial Strains

For this study, two model strains were selected, Gram (−) DH5α and Gram (+) ATCC6538P (American Type Culture Collection, Manassas, USA), and non-pathogenic strains characteristic of the oral cavity. In particular, the Gram (+) strains used was CECT 8313 (DSMZ, Germany), ATCC 35668 (DSMZ, Germany), (>99% identity with P4), and (100% identity with MBSb5a) isolated from the plaque of healthy patients. The Gram (−) bacterial strain used was ATCC 33277 (DSMZ, Germany).

#### 2.7.2 Antimicrobial Activity

Antimicrobial activity of CS and PCL was assessed by counting the cell viability of all of the selected Gram (−) and Gram (+) strains. CS, PCL, and hybrid samples were sectioned, weighed, sterilized under UV light for 8 h, and then incubated with bacterial cell suspension (5 × 10^5^ CFU/mL) for 4 h, adopting different concentrations of CS and PCL (10, 20, and 40 mg/mL) or hybrid scaffolds (75, 85, 95, and 105 mg/mL) and successively plated on LB agar plates. Bacterial cells have been adopted as a negative control. After 24 h of incubation, bacterial cell survival was determined by counting the number of colonies, according to recently published methods.^45,46^

#### 2.7.3 Determination of Minimal Inhibitory Concentration

The minimum inhibitory concentrations (MICs) of the CS, PCL, and hybrid scaffolds against all selected bacterial strains were determined by using the microdilution method in 96-well plates. A concentration of 5 × 10^5^ CFU/mL of each bacterial strain was added to 95 μL of Mueller–Hinton broth (CAM-HB; Fisher Scientific, Segrate, Italy), supplemented with CS, PCL or PCL/CS hybrid scaffolds at various concentrations (0–200 mg/mL).^47^ For , the MIC was determined using a similar method but in glass vials containing an anaerobic medium and atmosphere ideal for the growth of this bacterium (Tryptic Soy Broth supplemented with 0.5 μg/mL l-cysteine, 1 μg/mL menadione and 5 μg/mL hemin in an atmosphere of 85% N_2_, 10% H_2_ and 5% CO_2_).^48^ Gentamicin was used as a positive control for all of the bacterial strains. After overnight incubation at 37 °C, the MIC values were identified as the lowest concentrations of CS, PCL, and hybrid scaffolds that completely inhibited visible bacterial growth.

### 2.8 *In Vitro* Biological Analyses

#### 2.8.1 Cell Culture

PDLSCs, IV passage, were cultured in Dulbecco’s modified eagle medium high glucose (DMEM, Sigma-Aldrich, Darmstadt, Germany) supplemented with 10% (v/v) fetal bovine serum (FBS, Gibco, Thermo Fisher Scientific, Waltham, MA, USA), 200 mM l-glutamine [Euroclone, Pero (MI), Italy], and antibiotics (penicillin G sodium 100 U/mL, streptomycin 100 mg/mL, Euroclone, Pero (MI), Italy).

MG63 cells were maintained at 37 °C (5% CO_2_) in DMEM (Sigma-Aldrich, Darmstadt, Germany) supplemented with 10% FBS (Gibco, Thermo Fisher Scientific, Waltham, MA, USA), 1% penicillin/streptomycin [Euroclone, Pero (MI), Italy], 200 mM l-glutamine [Euroclone, Pero (MI), Italy] and 1% MEM non-essential amino acids solution (Sigma-Aldrich, Darmstadt, Germany).

The cells were incubated at 37 °C and 5% CO_2_ humidity and subcultured using trypsin/ethylene diamine tetraacetic acid (Sigma-Aldrich, Darmstadt, Germany).

All kinds of 3D scaffolds were properly treated before cell seeding using a Pen-Strep Solution (1000 mg/mL penicillin and 100 mg/mL streptomycin) for 2 h to supplement cell culture media for preventing bacterial contamination. The scaffolds were then cut to fit the cell culture plate wells.

#### 2.8.2 Cell Viability/Proliferation Assay

PDLSCs or, alternatively, MG63 were seeded onto the proposed scaffolds with a density of 5.0 × 10^4^ cells. To evaluate the proliferation and viability of PDLSCs or MG63 on neat 3D PCL or hybrid scaffolds, an Alamar Blue assay (AbD Serotec Ltd., Kidlington, UK) was employed. At 5, 7, and 14 days after cell seeding, the scaffolds were rinsed with PBS (Sigma-Aldrich, Milan, Italy), and 200 μL of DMEM without phenol red (HyClone, Cramlington, UK) containing 10% (v/v) Alamar Blue was added to each sample. The samples were incubated for 4 h in a controlled atmosphere at 5% CO_2_ and 37 °C. The supernatant was removed in a 96-well plate, and its absorbance was quantified by spectrophotometry at 570 and 595 nm. The levels of cell proliferation were expressed as a percentage of Alamar Blue reduction. The experiments were conducted three times in triplicate.

#### 2.8.3 Alkaline Phosphatase Activity

The osteogenic differentiation of PDLSCs and MG63 was evaluated in the case of 3D PCL and hybrid scaffolds using an early osteogenic differentiation marker, such as alkaline phosphatase (ALP). A specific enzymatic assay (SensoLyte pNPP Alkaline Phosphatase Assay Kit, AnaSpec Inc., Fremont, CA, USA) was used to evaluate the ALP activity. This assay was based on the phosphate-p-nitrophenyl substrate (pNPP). At 5, 7, and 14 days after seeding, the cells were washed twice in PBS and lysed in 1 mL of lysis buffer. After collection and centrifugation, the supernatant was mixed with an equal amount of pNPP working solution in a 96-well microplate. Mixtures were then incubated for 30 min at 37 °C. After a 30 min incubation with pNPP, the phosphatase was completely inhibited by NaOH and the pNPP liberating inorganic phosphate and the conjugate base of para-nitrophenol (pNP). The resulting phenolate was yellow with a maximal absorption at 405 nm. Measurements were compared to the alkaline phosphatase standard and normalized using the total protein amounts determined with the bicinchoninic acid (BCA) assay at 562 nm using a BCA protein assay kit (ThermoScientific, MA, USA).

#### 2.8.4 Statistical Analysis

All experiments were independently repeated three times. The data were represented as the mean value ± standard deviation.

*Two-way* ANOVA followed by Bonferroni test with multiple comparisons was performed using GraphPad Prism version 8.0.0 for Windows, GraphPad Software, San Diego, California USA, www.graphpad.com.

#### 2.8.5 Immunofluorescence Assays

Cell adhesion, morphology, and spatial distribution into the 3D structures were verified at 5, 7, and 14 days after cell seeding by confocal laser scanning microscopy (CLSM, Zeiss LSM 510/ConfoCor 2 system, Oberkochen, Germany). All samples were fixed with 4% paraformaldehyde for 1 h and treated with 0.1% Triton X-100 to permeabilize the cell membrane. Nuclei were stained with 4,6-diamidino-2-phenylindole (DAPI) dye (1 μg/mL), and actin filaments were stained with Phalloidin-AttoRho6G (100 μM), all by Sigma-Aldrich. Phalloidin fluorescence was collected in a spectral window of 500 to 530 nm. For DAPI stain acquisition, 720 nm excitation wavelength and 450–500 nm spectral window emission were used.

## 3 Results

### 3.1 Morphological Analyses

Typical SEM images of the neat 3D PCL scaffolds and hybrid scaffolds with dual porosity are shown in Figure 1. Results from SEM analysis highlighted that PCL structures were characterized by a fully interconnected pore network, thus confirming that the employed AM technique (*i.e.*, 3D fiber deposition) allows to obtain 3D porous structures with controlled and defined pore dimensions. SEM micrographs revealed a well-defined internal geometry with main square pores in the range 290.37 ± 35.35 μm for the fabricated PCL scaffolds. Furthermore, a network of smaller pores was well evident in the internal portion of the hybrid additive manufactured samples (Figure 1). CS network and hybrid scaffolds exhibited mean pore dimensions of 55.66 ± 13.33 and 40.12 ± 7.62 μm, respectively, with a quite regular pattern, also suggesting such conceived hybrid structures as potential candidates for hard tissue regeneration.^40^

**Figure 1:**
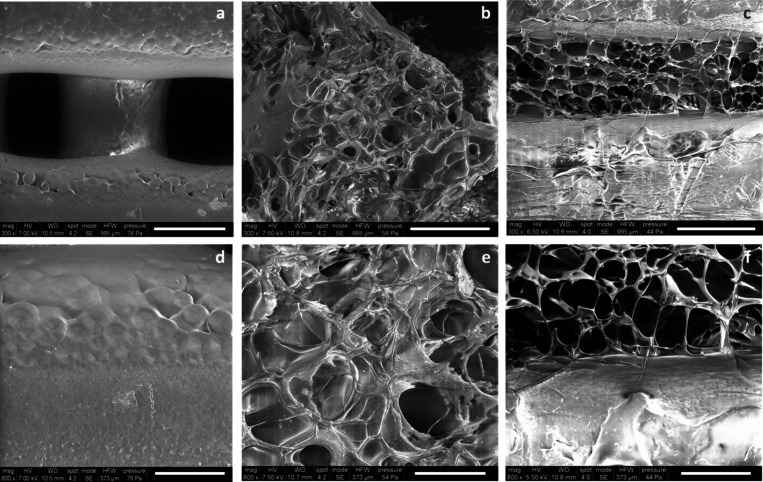
SEM images of the developed structures: PCL (a, d); CS (b, e); hybrid (c, f) at different magnifications. Scale bar: 300 μm (a–c) and 100 μm (d–f).

A highly porous structure with thin walls was observed via XCT measurements (Figure 2). Quantitative information about 3D scaffold porosity was collected via XCT postprocessing of the obtained data sets showing a mean % (v) of pores of about 62.09 ± 1.63 and 49.08 ± 0.87 for the PCL and hybrid scaffolds, respectively.

**Figure 2:**
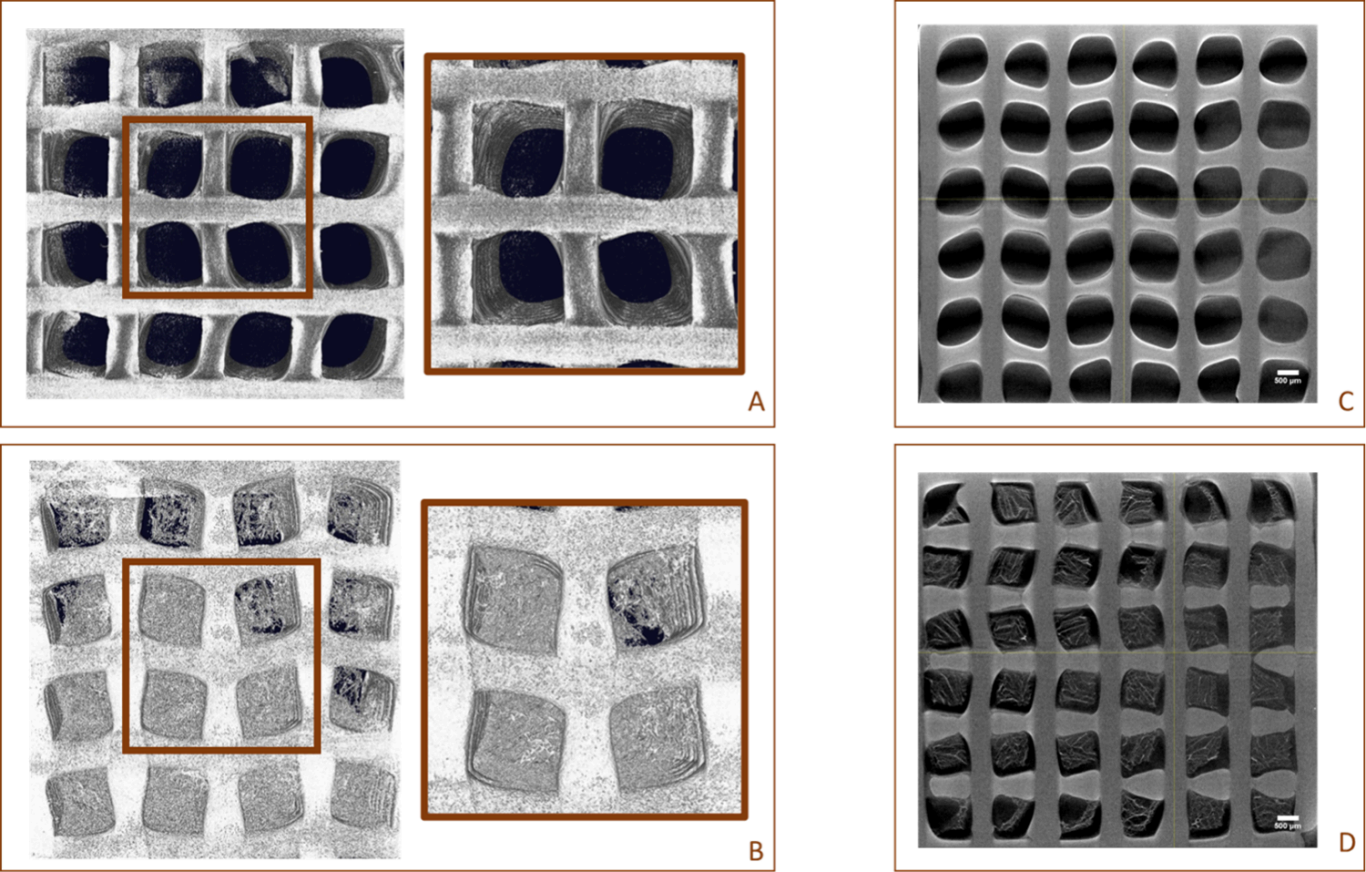
XCT analyses: 3D rendering of PCL (A) and hybrid (B) scaffolds. 2D slicing of PCL (C) and hybrid (D) scaffolds. Scale bar 500 μm.

### 3.2 Mechanical Measurements

Typical stress–strain curves for the developed 3D PCL or hybrid scaffolds are reported in Figure 3. Results from compression tests showed that the 3D PCL scaffold provided the main contribution to the overall mechanical behavior of the hybrid structures. In particular, the obtained findings show a mechanical behavior similar to that of a flexible foam.^44,49,50^ Figure 3A reports typical stress–strain curves from compression tests; a linear region is initially evident, followed by a region with lower stiffness. Finally, another stiff region of the stress–strain curve can be observed that resembles the densification region usually reported for flexible foams.^49^

**Figure 3:**
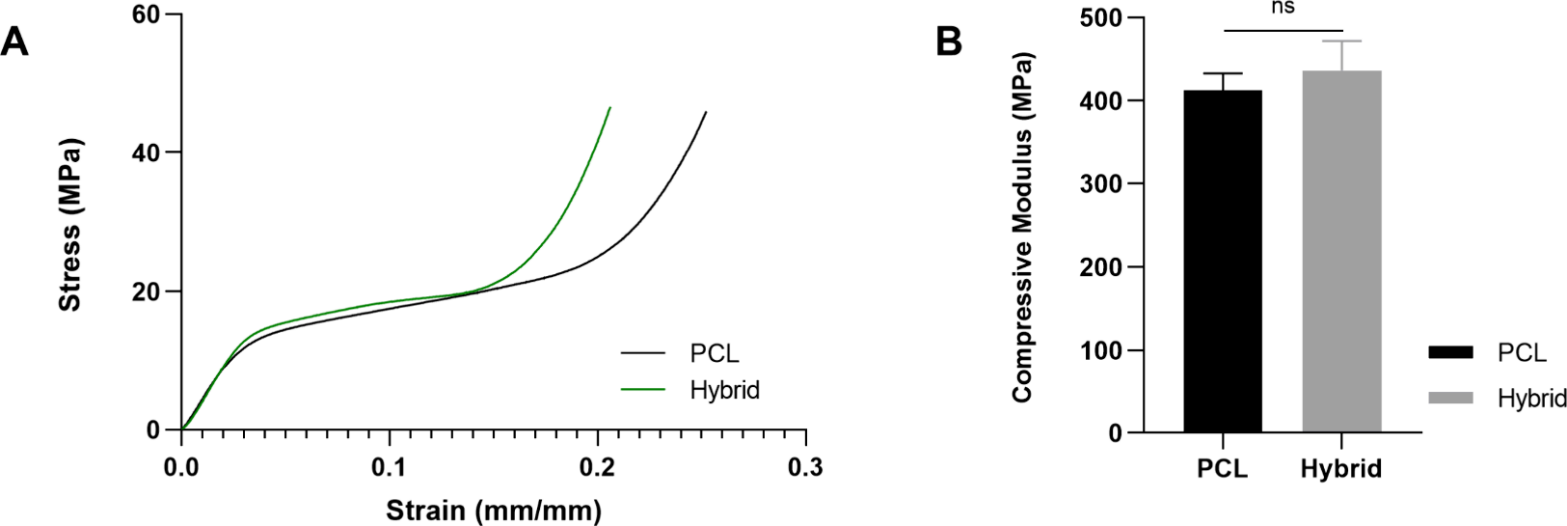
Typical stress–strain curves obtained from compression tests on 3D additive manufactured PCL and hybrid scaffolds (A); bar plot for compressive modulus (B) for 3D PCL and hybrid scaffolds. Results are reported as the mean value ± standard deviation. Statistical analysis of variance on mean values for compressive modulus was assessed by unpaired *t* tests (ns: not significant).

In particular, 3D PCL and hybrid scaffolds exhibited compressive moduli of 412.8 ± 20.2 and 436.5 ± 35.6 MPa, respectively. The addition of CS did not significantly improve the compressive modulus of the scaffolds, however providing a significant reduction of the strain (Figure 3B).

### 3.3 *In Vitro* Degradation

Water adsorption and degradation mechanism in terms of weight loss of proposed scaffolds were evaluated by gravimetric approach, where it can be assumed that the hydration mechanism is correlated with the water molecule penetration into the pore networks of the CS-based scaffolds.

Water uptake results (Figure 4A) highlighted that CS and hybrid scaffolds reached equilibrium after 24–36 h, with a more evident swelling capability for CS samples. Furthermore, neat CS scaffolds exhibited a faster degradation kinetic compared to hybrid or neat PCL scaffolds, as highlighted from weight loss measurements (Figures 4B). After 35 days (about 5 weeks) of incubation, about 60% weight loss can be noticed for CS scaffolds, while a weight loss of about 20 and 35% for PCL and hybrid structures, respectively, has been highlighted. PCL scaffolds exhibited a low degradation ratio, suggesting that the PCL structure may act as a barrier and provide additional support for new bone formation, while the rapid degradation of the CS scaffold could be responsible to activate new tissue formation during the eventual and sustained release of encapsulate drug/protein/genes, thus providing a novel strategy in bone tissue repair. The obtained findings are in agreement with the data reported by Dong et al. on similar structures.^23^

**Figure 4:**
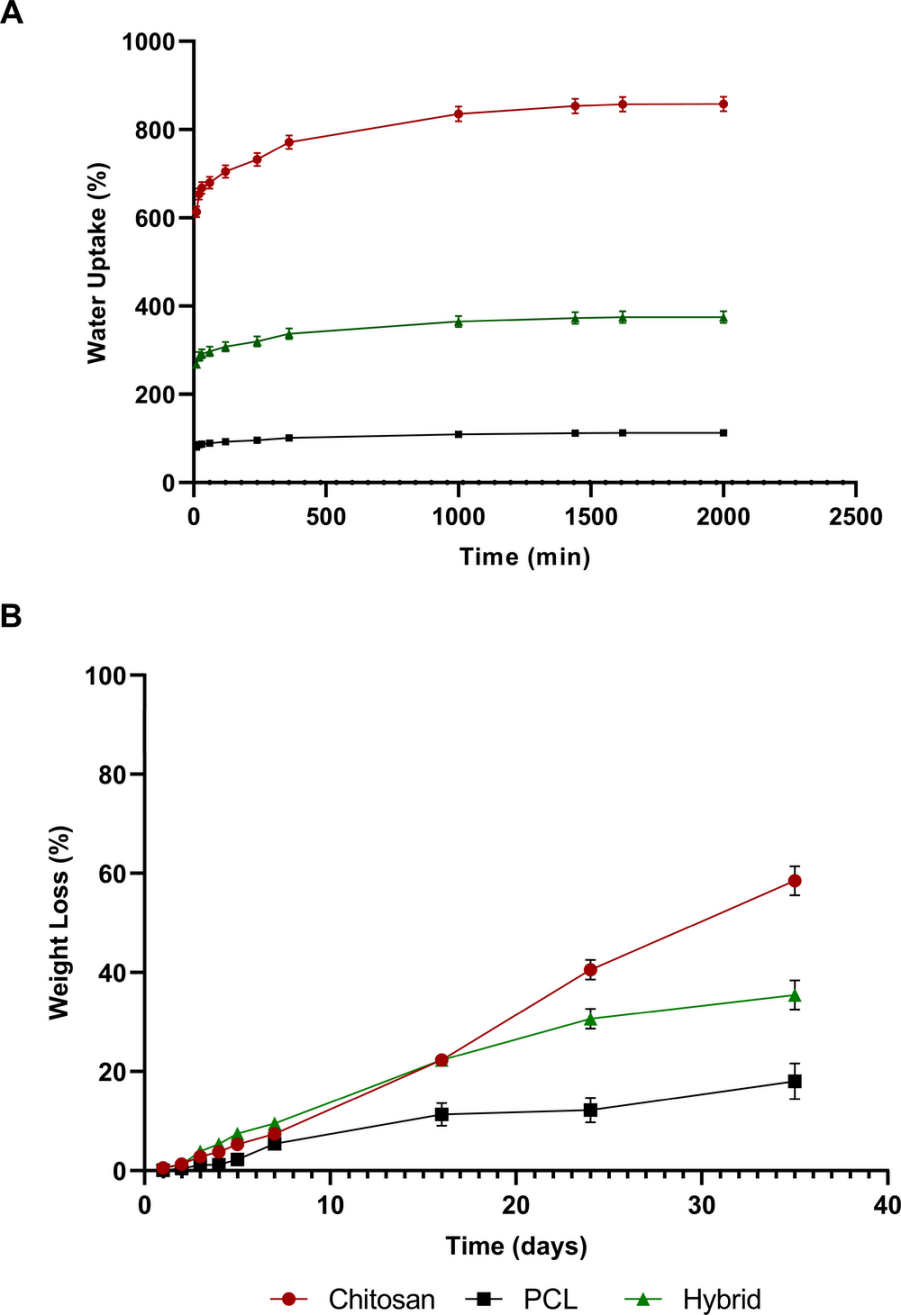
Results obtained from (a) water uptake and (b) weight loss on the proposed scaffolds.

### 3.4 Microbiological Analyses (Antibacterial Activity)

The antimicrobial properties of PCL, hybrid, and CS were evaluated using an antimicrobial activity test against the model strains and . The samples were sectioned, weighed, sterilized under UV light for 8 h, and then incubated with bacterial cells at concentrations of 10, 20, and 40 mg/mL (PCL and CS), 70, 80, 90, and 100 mg/mL (hybrid). As shown in Figure 5, the results demonstrated that CS was more effective, while PCL did not exhibit any antimicrobial activity, showing values comparable to the control. Notably, was more sensitive to CS, with bacterial survival inhibition of 50% even at 10 mg/mL. These findings are aligned with previous studies in the literature, which have shown that CS is more effective against Gram (+) strains.^51^ The hybrid system (Figure 5 panel B) shows some antimicrobial activity but at a higher concentration (70, 80, 90, and 100 mg/mL) than free CS, probably due to the presence of PCL.

**Figure 5:**
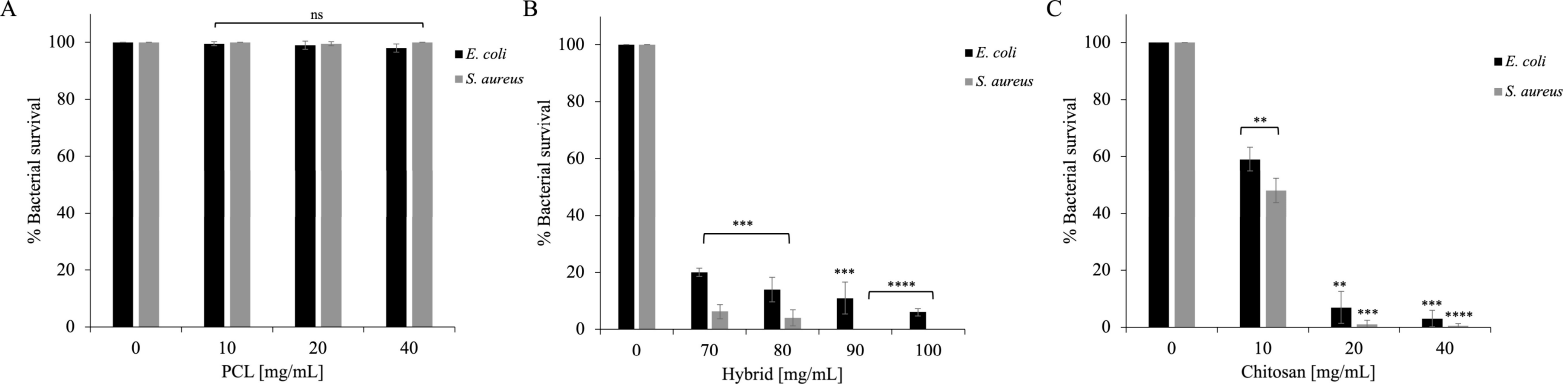
Antimicrobial activity of PCL (A), hybrid (B), and CS (C) against and . Tests were performed at concentrations of 10–20 and 40 mg/mL (A–C) and 70, 80, 90, and 100 mg/mL (B). The assays were performed in three independent experiments (*n* = 3). Statistical analysis was performed using a two-tailed paired *t* test (ns is not significant, **p ≤ 0.001, ***p ≤ 0.005, ****p ≤ 0.0001) versus control.

With the aim of developing hybrid PCL/CS scaffolds as an innovative and promising solution for enhancing oral cavity health, our antimicrobial properties were also tested against bacterial strains characteristic of the oral environment. Specifically, the Gram (+) strains , , , and were included in the study. The same concentrations of CS and PCL as in previous experiments were used; however, the growth conditions were adjusted using gas-pack systems as these bacteria are microaerophilic. As shown in Figure 6C, all strains tested demonstrated high sensitivity to 40 mg/mL CS. In particular, was found to be the most sensitive, which is particularly interesting given its well-known role in dental plaque, periodontitis, and formation and development of caries.^52,53^ Once again, PCL did not exhibit any antimicrobial activity, displaying values comparable to those of the control (Figure 6A).

**Figure 6:**
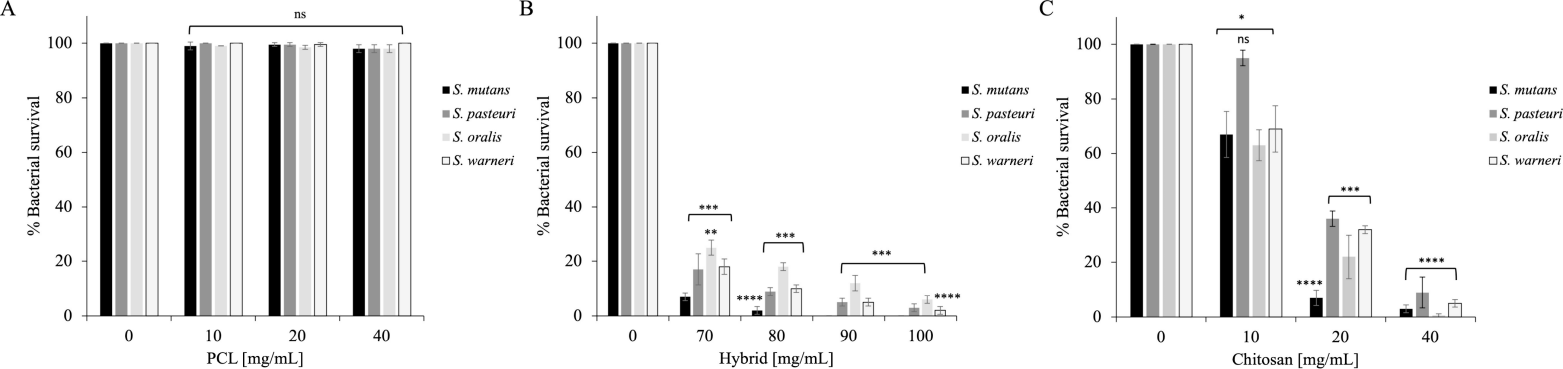
Antimicrobial activity of PCL (A), hybrid (B), and CS (C) against , , , and . Tests were performed at concentrations of 10, 20, and 40 mg/mL (A–C) and 70, 80, 90, and 100 mg/mL (B). The assays were performed in three independent experiments (*n* = 3). Statistical analysis was performed using a two-tailed paired *t* test (ns is not significant, *p ≤ 0,05; **p ≤ 0.001, ***p ≤ 0.005, ****p ≤ 0.0001) versus control.

As mentioned above, PCL represents a biodegradable and biocompatible polymer widely adopted in medical applications, tissue engineering, and controlled drug release.^54^ However, it lacks intrinsic antimicrobial properties.^63^ PCL does not exhibit intrinsic antimicrobial activity, as confirmed by our findings and supported by the literature. This is due to its chemical structure: PCL is a hydrophobic, aliphatic polyester that lacks functional groups capable of interacting with microbial membranes or interfering with cellular processes.^55^ While PCL is highly biocompatible and extensively used in tissue engineering and controlled drug delivery, its chemical inertness renders it microbiologically inactive.

In contrast, the antimicrobial activity of CS is attributed to several well-documented mechanisms, mainly related to its polycationic nature. Under acidic or neutral pH conditions, the amino groups (−NH_2_) present on the CS backbone become protonated (−NH_3_^+^), enabling electrostatic interactions with negatively charged bacterial cell wall components, such as lipopolysaccharides in Gram (−) bacteria and teichoic acids in Gram (+) bacteria.^56^ These interactions can lead to increased membrane permeability, resulting in leakage of intracellular components and cell death.^57^ Furthermore, CS is known to chelate metals and essential nutrients, disrupting enzymatic activity and microbial metabolism.^58^ It can also form a film on microbial surfaces, acting as a physical barrier that limits nutrient uptake and inhibits growth.^59^ These multifunctional mechanisms explain its broad-spectrum activity and the particularly high sensitivity observed in Gram (+) strains such as and oral pathogens such as . To impart antimicrobial activity to PCL-based devices or materials, it is often necessary to combine PCL with other antimicrobial agents.^60^ This led us to integrate it with CS.

The antimicrobial properties of the PCL/CS hybrid scaffolds were assessed using an antimicrobial activity assay. The scaffold was sectioned, weighed, and UV sterilized as before. It was then incubated with bacterial cells at concentrations of 70, 80, 90, and 100 mg/mL. After 24 h, dose–response curves were generated by counting individual colonies. Specifically, results highlighted in Figure 6B confirm the higher sensitivity of the oral strains , , and , which show the highest sensitivity to the hybrid scaffold.

The MIC for bacterial growth of CS, PCL and the hybrid scaffold was determined using the microdilution method. Table 3 presents the MIC values obtained. Consistently with previous experiments, the lowest MIC values for CS (18–45 mg/mL) were recorded against , , , and . In contrast, MIC values for PCL could not be determined, as they were all above 200 mg/mL. In line with the results obtained thus far, the hybrid system shows slightly higher MIC values than free CS. This is expected as PCL likely partially masks the antimicrobial action of CS. To further investigate the antimicrobial properties of CS against oral cavity strains, the MIC was also evaluated against . An anaerobic vial growth system was used to facilitate the growth of this bacterium. The results showed an MIC value of 62 mg/mL against this bacterium. This result is promising given the involvement of in periodontitis. Additionally, the possibility to locally target the bacteria could be useful in lowering the release of its virulence factors, including gingipains, which are implicated in AD.^9^

**Table 3:** Evaluation of the MIC Values (Expressed as Mean ± Standard Deviation) of CS, PCL, and the CS/PCL Hybrid against All Selected Bacterial Strains (MIC Values Determined from At Least Three Independent Experiments)

	MIC_100_ [mg/mL] ± SD		
strains	CS	PCL	hybrid
*E. coli*	42 ± 1.3	>200	120 ± 2.5
*S. aureus*	18 ± 0.9	>200	100 ± 1.6
*S. oralis*	50 ± 1.4	>200	120 ± 1.8
*S. mutans*	42 ± 1.3	>200	98 ± 1.4
*S. warneri*	45 ± 1.4	>200	110 ± 2.5
*S. pasteuri*	40 ± 1.2	>200	110 ± 2.6
*P. gingivalis*	62 ± 5	>200	142 ± 8

### 3.5 *In Vitro* Biological Analyses

#### 3.5.1 Cell Viability/Proliferation Assay

The Alamar Blue assay was carried out to quantitatively investigate the viability of PDLSCs or MG63 cells seeded onto neat 3D PCL or hybrid scaffolds. As shown in Figure 7A,B, cells seeded on the proposed scaffolds highlighted a good and uniform viability over the culture time. For both cell lines, a peak at 7 days from seedings can be observed. The percentage of reduction, and therefore cell proliferation, appeared to be higher in both cell lines seeded on hybrid structures, highlighting a better microenvironment for initial and sustained cell retention.

**Figure 7:**
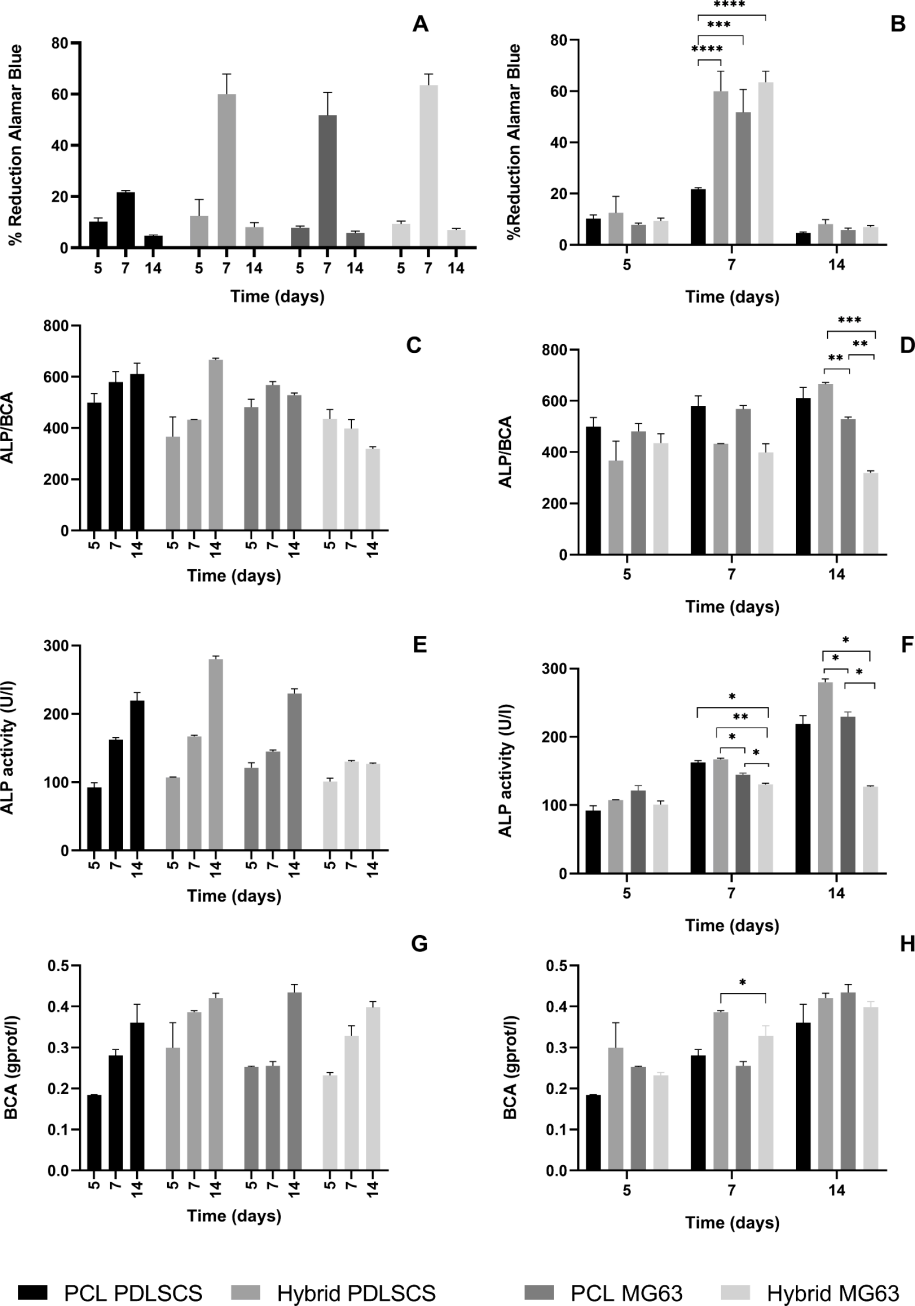
*In vitro* biological assays. Percentage of Alamar Blue reduction at different time points for PDLSCs and MG63 seeded on PCL or hybrid scaffolds (A, B). Results are reported as mean value ± standard deviation. Statistical analysis of variance on mean values was assessed by *two-way* ANOVA (ns, *p < 0.1, **p < 0.01, ***p < 0.001, ****p < 0.0001). ALP activity for PDLSCs and MG63 seeded on PCL or hybrid scaffolds at 5, 7, and 14 days. ALP activity (E, F) has been normalized with BCA (G, H) to obtain the ALP/BCA (C, D). Results are reported as mean value ± standard deviation. Statistical analysis of variance on mean values was assessed by *two-way* ANOVA followed by Bonferroni test with multiple comparisons. (ns, *p < 0.1, **p < 0.01, ***p < 0.001, ****p < 0.0001).

#### 3.5.2 ALP Activity

Cell osteogenic differentiation analyses were performed using ALP as an early osteogenic marker and normalized with the BCA protein assay (Figure 7).

ALP levels normalized with BCA are shown in Figure 7C,D, highlighting that the ALP expression seems to be higher for PDLSCs seeded on hybrid structures at 14 days from seeding. Mean values are statistically different if compared to MG63 seeded on both PCL or hybrid structure (**p < 0.01 and ***p < 0.001, respectively). These results are in line with the results reported by recent literature studies.^61,62^

At the investigated time points, the ALP expression seems to be significantly higher for PDLSCs when compared to MG63. This finding validates current studies which identify PDLSCs as an innovative source of stem cells used in regenerating treatments based on tissue engineering.^63^ Due to their high self-renewal capacity and very similar characteristics to mesenchymal stem cells, PDLSCs seem to better interact with hybrid samples, thus confirming that the proposed approach represents a promising strategy for hard tissue regeneration. The trend in the results for ALP expression is consistent with literature data and evidenced by different studies like those conducted by Sun and Tsai.^64,65^

#### 3.5.3 CLSM

Cell morphology was analyzed using CLSM to assess the ability of PDLSCs and MG63 cells to adhere to PCL and hybrid scaffolds (Figure 8) and to observe their morphological adaptation over the culture period. CLSM images confirmed the quantitative findings obtained with the Alamar Blue assay. Cells adhered uniformly to all of the tested scaffolds. Over time, cell morphology evolved from a predominantly rounded shape to a more elongated and spread appearance, suggesting enhanced cell–material and cell–cell interactions. This behavior may be attributed to the synergistic effect of the surface chemistry and topography of the additively manufactured scaffolds.

**Figure 8:**
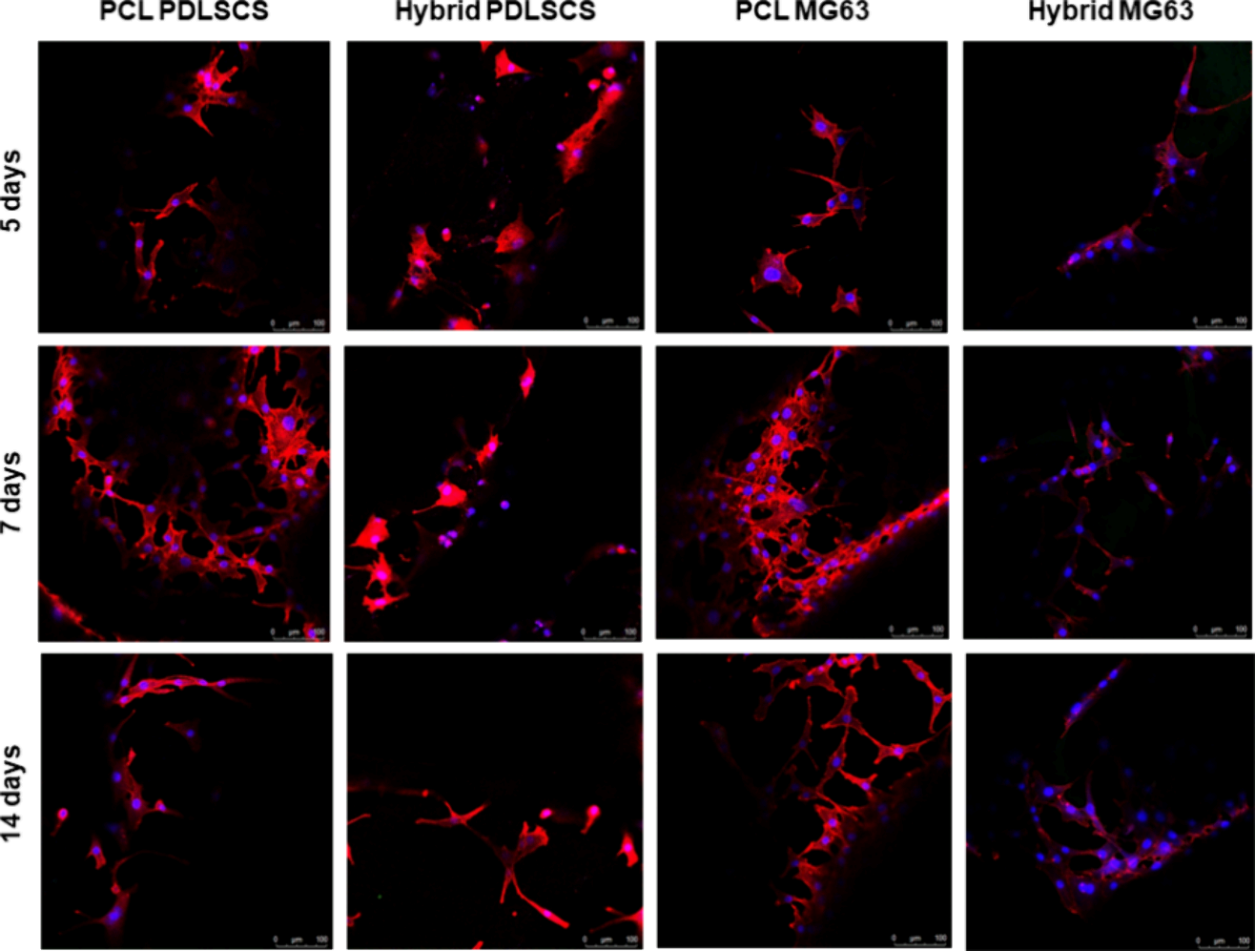
Results from CLSM analysis at different time points on 3D PCL or hybrid scaffolds with PDLSCs or MG63. (Column I) From top to bottom, PCL PDLSCs at 5, 7, and 14 days after cell seeding. (Column II) From top to bottom, hybrid PDLSCs at 5, 7, and 14 days from seeding. (Column III) From top to bottom PCL MG63 at 5, 7, and 14 days after cell seeding. (Column IV) Hybrid MG63 at 5, 7, and 14 days after cell seeding. Nuclei were stained with the DAPI dye in blue, and actin filaments were stained with Phalloidin-AttoRho6G in red. Scale bar: 100 μm.

## 4 Discussion

An integrated approach in designing 3D additive manufactured hybrid scaffolds was proposed in the present study. Specifically, 3D-printed and morphologically controlled PCL scaffolds with appropriate mechanical features were combined with CS porous networks, thus obtaining PCL/CS hybrid scaffolds with the aim of combining different polymer sources, also benefiting from the advantages of both conventional and AM technologies toward multifunctional structures with improved structural and functional features. The final aim to be pursued relies on the possibility to tailor degradation times of the different compartments of the hybrid structures, thus adopting the proposed strategy to trigger local drug release over degradation time.

Morphological analyses from SEM and XCT analyses confirmed that PCL structures were characterized by a 100% interconnected pore network with a well-defined internal geometry with main square pores in the range of 290.37 ± 35.35 μm. Furthermore, the neat and internal CS network exhibited a network of smaller pores characterized by mean pore dimensions of 55.66 ± 13.33 and 40.12 ± 7.62 μm, respectively, with a quite regular pattern.

Mechanical performances were evaluated through compression tests performed on both polymeric and hybrid structures. The obtained results suggested a mechanical behavior similar to that of flexible foam.^44,49,50^

The inclusion of the CS network did not alter the compressive modulus of the 3D PCL scaffolds, however, improving the dimensional stability of the developed structure (*i.e.*, strain reduction).

Swelling and degradation behavior have been evaluated by gravimetric approach, whereas degradation phenomena occurring to the external polymeric structure have been analyzed in the recent literature. Regarding the swelling and degradation behavior of the inner CS porous network, the hydration mechanism can be assumed as related to the water molecule penetration into the pore networks of the CS-based scaffolds. Water uptake results have highlighted that all samples (PCL and hybrid scaffolds) reached equilibrium after 24–36 h, while the weight loss measurements evidenced that the neat CS structures exhibited a higher degradation rate compared to PCL-based scaffolds. A 60% weight loss was observed for CS after about 35 days, while weight losses of about 20 and 35% were found for PCL and hybrid structures, respectively.

Alamar Blue assay performed to investigate the viability of PDLSCs or MG63 cells seeded onto PCL and hybrid porous scaffolds has shown that cells seeded in the different kinds of scaffolds maintain a good and uniform viability over the culture time. For both cell lines, a peak after 7 days after cell seeding and a decrease in cell growth at 14 days can be observed. The percentage of reduction, and therefore cell proliferation, appeared to be higher for PDLSCs seeded on hybrid structures than for the other analyzed samples, probably due to the increased intrinsic self-renewal capacity of PDLSCs and more surface area exhibited by the hybrid scaffolds.^66^

Cell osteogenic differentiation was analyzed by means of ALP expression as an early osteogenic marker normalized with the BCA protein assay. Different results were obtained in terms of ALP expression of PDLSCs depending on the sample composition. ALP levels normalized with BCA in PDLSCs seeded hybrid scaffolds highlighted better results compared to the other analyzed conditions. Furthermore, at 14 days from seeding, ALP levels seem higher for all analyzed samples, with mean values statistically different compared to MG63 seeded on both PCL and hybrid structure (**p < 0.01 and ***p < 0.001, respectively). This latest result is in line with the results reported by recent literature studies,^61,62,64,65^ also confirming recent evidence supporting PDLSCs as an innovative source of stem cells used in tissue regeneration treatments.^63^ Due to their high self-renewal capacity and very similar characteristics to mesenchymal stem cells, PDLSCs seem to better interact with hybrid samples, thus confirming that the proposed approach represents a promising strategy for the purposes of the present work.

## 5 Conclusions

The current research may be considered as a first step of a future complex work with the aim of designing 3D hybrid scaffolds characterized by dual porosity for tissue-engineering purposes, adopting a proper combination of different material chemistry, technological approaches, design methods, and analyses. The possibility of conceiving an internal porous CS network improves the dimensional and mechanical stabilities of the proposed 3D additive manufactured hybrid scaffolds over time, also highlighting that they could be responsible for the exhibition of intrinsic signals for cell retention and differentiation. Recent literature has explored the morphology and mechanical features of the different components of the periodontium. Naveh et al.^67^ classified the components of the tooth–periodontal ligament (PDL)–bone complex according to their stiffnesses or elastic moduli, evidencing values ranging from kPa to few MPa for PDL and up to hundreds of GPa for peritubular dentine and enamel. On the other hand, Ho et al.^68^ reported values of reduced modulus (*E*_r_) for PDL-bone obtained via nanoindentation measurements ranging from 10 to 50 MPa for PDL, from 0.2 to 9.6 GPa for bone, and from 1.1 to 8.3 GPa for cementum, also trying to correlate the obtained values with the macroscale function of the bone-tooth complex.

Our approach aimed to achieve a balanced modulus sufficiently high to support mechanical stability and maintain structural integrity during implantation, adequate to impair cellular response or integration with surrounding soft tissues. The current design, with a porosity of 50–60% and pore size around 300 μm, provides a compressive modulus of 412.8 ± 20.2 and 436.5 ± 35.6 MPa for 3D PCL and hybrid scaffolds, respectively, suitable for supporting the regeneration of both periodontal ligament and adjacent mineralized tissues while also being tunable depending on the specific application site within the periodontal complex.

The synergistic contribution of material chemistry and scaffold architecture could be strategically tuned to optimize the final structure as a CS-based reservoir for the controlled and localized release of drugs, proteins, or genes—aimed at enhancing periodontal regeneration and/or augmentation. Leveraging the intrinsic antimicrobial properties of the internal porous CS network combined with the specific chemical characteristics of the selected materials, this platform holds significant promise. Future studies will further explore the incorporation of targeted antibacterial cues to enhance efficacy against specific periodontal pathogens. Recent findings suggested antimicrobial peptides (AMPs) as promising molecules limiting the use of conventional antibiotics, exhibiting antimicrobial, antiviral, and/or antifungal activities, at the same time improving immunomodulation and inhibition of lipopolysaccharide (LPS)-induced inflammation.^69^ In particular, SQQ30 AMP has been proposed as a promising antimicrobial agent evidencing antimicrobial, LPS binding, and immunomodulatory features as well as its role in oxidative stress.^46^

In this scenario, the present work would like to establish the basis for a complex and customized hybrid structure design with improved features as a novel approach in periodontal repair, also evidencing that the optimization strategy for the complex tissue composed of gingiva, cementum, periodontal ligament, and alveolar bone still requires several research efforts also involving structured preclinical studies, case reports, and clinical trials. While the proposed scaffold design demonstrates promising *in vitro* results in terms of biocompatibility, cellular response, and structural performance, a key limitation of this study is the lack of *in vivo* validation. Future investigations will focus on graded porosity and compartmentalized scaffold optimization with improved antimicrobial features and their validation in preclinical models to assess the features of the scaffolds within the complex physiological environment, particularly in terms of tissue integration, inflammatory response, and long-term functional regeneration, to confirm the translational potential of the proposed system for clinical application in periodontal tissue repair.

## Data Availability

Data will be made available on request.
